# Osteointegration of porous absorbable bone substitutes: A systematic review of the literature

**DOI:** 10.6061/clinics/2017(07)10

**Published:** 2017-07

**Authors:** Maria Júlia Escanhoela Paulo, Mariana Avelino dos Santos, Bruno Cimatti, Nelson Fabrício Gava, Marcelo Riberto, Edgard Eduard Engel

**Affiliations:** Departamento de Biomecanica, Medicina e Reabilitacao do Aparelho Locomotor, Faculdade de Medicina de Ribeirao Preto, Universidade de Sao Paulo, Ribeirao Preto, SP, BR

**Keywords:** Biomaterials, Osteointegration, Systematic Review, Bone Substitute

## Abstract

Biomaterials’ structural characteristics and the addition of osteoinductors influence the osteointegration capacity of bone substitutes. This study aims to identify the characteristics of porous and resorbable bone substitutes that influence new bone formation. An Internet search for studies reporting new bone formation rates in bone defects filled with porous and resorbable substitutes was performed in duplicate using the PubMed, Web of Science, Scielo, and University of São Paulo Digital Library databases. Metaphyseal or calvarial bone defects 4 to 10 mm in diameter from various animal models were selected. New bone formation rates were collected from the histomorphometry or micro-CT data. The following variables were analyzed: animal model, bone region, defect diameter, follow-up time after implantation, basic substitute material, osteoinductor addition, pore size and porosity. Of 3,266 initially identified articles, 15 articles describing 32 experimental groups met the inclusion criteria. There were no differences between the groups in the experimental model characteristics, except for the follow-up time, which showed a very weak to moderate correlation with the rate of new bone formation. In terms of the biomaterial and structural characteristics, only porosity showed a significant influence on the rate of new bone formation. Higher porosity is related to higher new bone formation rates. The influence of other characteristics could not be identified, possibly due to the large variety of experimental models and methodologies used to estimate new bone formation rates. We suggest the inclusion of standard control groups in future experimental studies to compare biomaterials.

## INTRODUCTION

The use of autografts for the treatment of bone defects has well-known restrictions, including limited availability and donor site morbidity 1. This fact has sparked an intense search for bone substitutes of different compositions and structural conformations 2. Some are already available on the market, whereas many still await evidence attesting their capacity for osteointegration and consequent commercial viability.

A large number of new materials and material combinations have been developed. Structural characteristics have also been improved. The presence of pores significantly increases the osteointegration capacity, whereas solid biomaterials tend to form a fibrosis layer on the surface 3. Furthermore, the presence of pores allows fluid circulation inside the biomaterial, accelerates absorption of absorbable biomaterials and decreases the peak temperature of cements during setting 4,5. Most authors believe that pore size, porosity and interconnection of pores enhances new bone ingrowth. The ideal magnitude of these characteristics, however, has not yet been established 5-7. Progressive reabsorption and replacement of the biomaterial by normal bone is also considered an advantageous property because inert substitutes affect bone remodeling and can compromise its structure and mechanical resistance 8. The addition of growth factors and other osteoinductive factors seems to increase osteointegration, but conflicting data also exist 9.

Comparing the many combinations of materials is a demanding task, and the use of many different analysis methods makes comparisons even more challenging. Computed microtomography (micro-CT) and histomorphometry (HMM) have frequently been used to quantify new bone formation (NBF) in bone defects created in animal models and inside the porous biomaterial 10.

The aim of this study was to identify the chemical and structural characteristics that influence the capacity of new bone formation of porous and absorbable bone substitutes implanted in animal models using a systematic review.

### Methodology

A systematic review was performed to evaluate how specific characteristics of porous and absorbable bone substitutes used to fill bone defects in experimental *in vivo* studies affects the capacity of new bone formation using micro-CT or HMM quantifications.

### Search strategy

An electronic search was independently performed by two researchers (MJEP and MAS) between July 2014 and February 2015 without restrictions on the publication date. The following databases were used: PubMed, Web of Science, Scielo, and Theses and Dissertations of the University of São Paulo Digital Library. Only articles written in English or Portuguese were selected. The following search term combinations were used: (bone substitute AND porous) OR (bone substitute AND cancellous) OR (bone substitute AND spongy) OR (bone substitute AND pore) OR (cement AND porous) OR (cement AND cancellous) OR (cement AND spongy) OR (cement AND pore) OR (bone cement AND porous) OR (bone cement AND cancellous) OR (bone cement AND spongy) OR (bone cement AND pore).

### Article selection strategy

**Figure 1:**
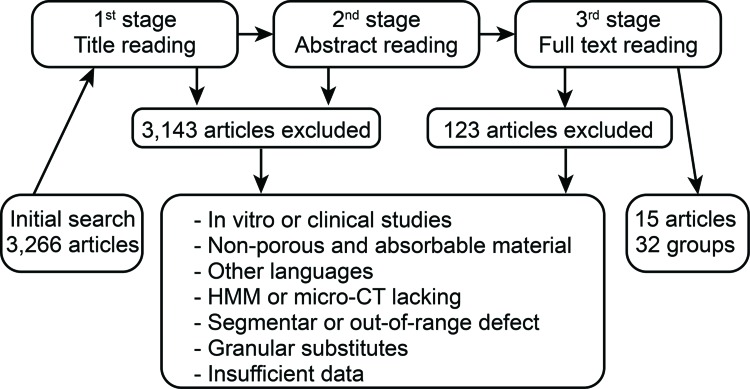
Search strategy.

Two researchers (MJEP and MAS) independently performed the article selection based on the eligibility criteria by reading the titles, summaries or full text, according to the search strategy (Figure 1). Consensus meetings, with the participation of a third researcher (EEE), were utilized to resolve conflict situations. Some articles were included from the reference lists of the selected articles.

The experimental groups of each article that met eligibility criteria were analyzed independently; therefore, each article could have more than one group.

### Eligibility criteria

Inclusion criteria:

1. Experimental, *in vivo* studies in animal models;
2. Orificial defects, 4.0 mm to 10.0 mm in diameter, produced by curettage or drilling of holes;
3. Implantation of porous and resorbable bone substitutes in the form of premolded blocks or cement with a clear description of the composition, porosity and pore size;
4. NBF indicated as a rate according to HMM or micro-CT data.

Exclusion criteria:

1. Clinical trials, implantation in humans or *in vitro* studies;
2. Insufficient description of the substitute characteristics, methodology or results;
3. Non-porous or non-absorbable substitutes;
4. Presentation in granular form. This form was excluded due to interference with the porosity and pore size of the substitute.

### Variables

The end point variable was the rate of new bone formation (NBF), which was based on the histomorphometry (HMM) or micro-CT analysis data. The remaining dependent variables were as follows: animal model, bone region of the defect, diameter of the defect (in mm), follow-up time after implantation (in weeks), substitute basic material (calcium phosphate, hydroxyapatite, bioglass, etc.), osteoinductor addition (fibroblast growth factor (FGF), BMP or bone marrow mesenchymal stem cells (BMSCs)), maximum pore size and porosity.

### Presentation of results

The NBF rates are described as the means, maximum and minimum values. The categorical variables and maximum defect size were grouped and compared using the Kruskal-Wallis test. The defect size, follow-up time and porosity were correlated to NBR using the Spearman’s correlation coefficient. PASW software version 17 (IBM SPSS, Armonk, USA) was used for the data analysis and the level of significance was set as 5%.

## RESULTS

The initial search identified 3,266 studies. Figure 1 illustrates the selection flow. A total of 3,143 articles were excluded because the biomaterial characteristics, experimental model, NBF measurement or article language were not eligible. Another 108 articles were excluded due to insufficient data or incomplete description of the biomaterial, methodology or defect characteristics. From the remaining 15 articles, 32 experimental groups with different implanted bone substitutes were considered for the analysis (Table 1).

**Figure 2:**
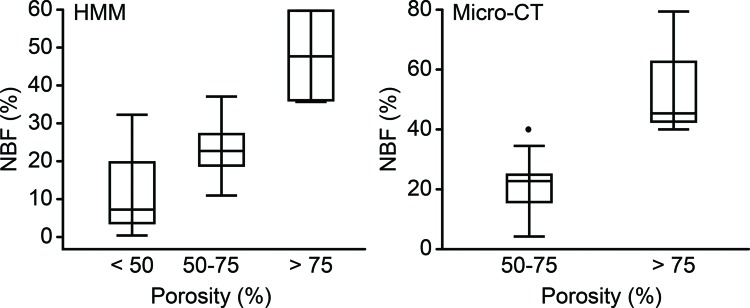
Boxplot of the NBR rates of the different porosity groups according to the analysis method (HMM or micro-CT). NBF, new bone formation; HMM, histomorphometry. *p*-values from Kruskal–Wallis test.

HMM was used more frequently than micro-CT for NBF quantification (20 groups, 62.5%). In both analysis methods, no significant differences were found when the NBF means were grouped according to the animal model and bone region (Table 2). The basic materials of the bone substitutes could not be compared because many of the material groups became too small due to uneven distribution of the experimental groups. The addition of osteoinductors to the bone substitute did not result in a significant increase in NBF. In addition, the influence of the pore size on NBF could not be detected. There was no correlation between the defect size and NBF in the HMM analysis (r=–0.181, *p*=0.446) and micro-CT analysis (r=0.103, *p*=0.751). NBR showed a moderate correlation to the follow-up time in the HMM analysis (r=0.441, *p*=0.052) but a weak correlation in the micro-CT analysis (r=0.122, *p*=0.705). NBF was significantly influenced by porosity (Figure 2).

## DISCUSSSION

The search for bone substitutes is a contemporary and relevant subject 9. The large number of studies that present new biomaterials or the further development of known biomaterials confirms this statement. Prior to February 2015, 3,266 studies were published describing porous and absorbable bone substitutes filling bone defects in experimental animals.

A large number of materials have been developed and tested, separately or combined, to increase the capacity of new bone formation. This continuous and effervescent development hinders the classification of these materials 9. In this review, we aimed to classify these materials according to the basic material used in the production of the bone substitute; however, there are overlaps between groups and many materials that are classified into the same group are not always similar. Most of the substitutes contained HA or other types of CPs or a combination of them as the basic material. The accumulated experience on modulating the resorption rate and mechanical resistance of these compounds make this an attractive combination 10,18. The number of other materials was too small to allow a statistical analysis.

The influence of the other chemical and structural characteristics of the biomaterials had to be very large to be detected in such a heterogeneous sample. For this reason, it was not possible to identify differences in the capacity of new bone formation by comparing the different groups of experimental animals, the sizes and locations of bone defects, the association with osteoinducers and the pore sizes. The follow-up time presented a weak to moderate correlation to NBF, depending on the analysis method (HMM or micro-CT). However, the porosity presented significant differences, suggesting that this parameter has a strong impact on the NBF rate.

Despite the large number, few studies could be included due to the significant variation in the presentation of the bone substitutes and experimental models. In addition, the NBF assessment method varied greatly, making comparison unsuitable. In this review, we used the two most cited methods, which, according to some articles, do not present comparable values but are related 10,25,26. Therefore, the HMM and micro-CT results were analyzed separately.

Although a substantial effort has been made by the scientific community in recent years to improve bone substitutes, some skepticism still exists regarding the effectiveness of the currently available biomaterials to cure critical size bone defects 27. The wide variety and geometrical shapes of bone materials indicates that many questions remain to be answered. More research is necessary to better understand how NBF can be increased.

This review demonstrates that the lack of standardization of NBF analyses has hampered the comparison of the various types of porous and absorbable bone substitutes. Even when limiting the evaluation to two types of quantitative analyses for NBF, it was not possible to obtain accurate results. A better description of the analyzed region of interest (ROI) by each method could offer more precise data interpretation, and consequently, a more consistent comparison with other similar studies. Additionally, the inclusion of standard control groups with autografts or a commonly used substitute would allow the calibration of the results and the comparison of many different study groups.

We conclude that porosity has a high impact on NBF rates and that the current lack of standardized analysis methods and the broad variety of experimental models makes identifying the chemical and structural characteristics that provide a greater capacity for NBF almost impossible. We suggest that standard control groups should always be used to allow for a better comparison of the results.

## AUTHOR CONTRIBUTIONS

Paulo MJ and dos Santos MA conducted the electronic search and data collection. Cimatti B and Gava NF reviewed the electronic search and data collection. Riberto M revised the manuscript and conducted the data analysis. Engel EE wrote the manuscript, reviewed the data analysis and supervised the study.

## Figures and Tables

**Table 1:** Characteristics of the experimental groups.

Author	Animal	Region	Defect size (mm)	Follow-up (weeks)	Basic material	Osteoinductor	Maximum pore size (μm)	Porosity (%)	Evaluation method	NBF (%)
Del Real et al. 11	Goat	Metaphysis	6.3	10	CP	-	100	47.0	HMM	0.0
Del Real et al., 2002	Goat	Metaphysis	6.3	10	CP	-	100	59.0	HMM	20.0
Hing et al. 12	Rabbit	Metaphysis	4.5	26	HA	-	350	60.0	HMM	28.0
Hasegawa et al. 13	Rabbit	Metaphysis	6.0	26	HA	-	480	70.0	HMM	27.0
Hasegawa et al. 13	Rabbit	Metaphysis	6.0	26	HA	-	200	70.0	HMM	23.0
von Doernberg et al. 14	Goat	Metaphysis	8.0	24	CP	-	150	75.0	HMM	22.0
von Doernberg et al. 14	Goat	Metaphysis	8.0	24	CP	-	260	75.0	HMM	37.0
von Doernberg et al. 14	Goat	Metaphysis	8.0	24	CP	-	510	75.0	HMM	25.0
von Doernberg et al. 14	Goat	Metaphysis	8.0	24	CP	-	1220	75.0	HMM	20.0
Kroese-Deutman et al. 15	Rabbit	Calvaria	6.0	12	CP	-	150	71.0	HMM	17.0
Kroese-Deutman et al. 15	Rabbit	Calvaria	9.0	12	CP	-	150	74.0	HMM	18.0
Xu et al. 16	Rabbit	Calvaria	10.0	16	CS	-	400	74.9	HMM	28.4
Xu et al. 16	Rabbit	Calvaria	10.0	16	CP	-	400	72.3	HMM	18.8
Tang et al. 3	Rabbit	Metaphysis	5.5	16	HA	-	394	36.0	HMM	32.2
Keiichi et al. 17	Rat	Metaphysis	4.3	12	HA	-	500	52.0	Micro-CT	20.4
Keiichi et al. 17	Rat	Metaphysis	4.3	12	HA	FGF	500	52.0	Micro-CT	28.7
Keiichi et al. 17	Rat	Metaphysis	4.3	12	HA	FGF	500	52.0	Micro-CT	28.5
Calvo-Guirado et al. 18	Rabbit	Metaphysis	6.0	8	HA+CP	-	450	95.0	HMM	60.0
Okanoue et al. 19	Rabbit	Metaphysis	5.0	12	HA	-	300	85.0	Micro-CT	43.0
Okanoue et al. 19	Rabbit	Metaphysis	5.0	12	CP	-	400	75.0	Micro-CT	15.6
Su et al. 20	Rabbit	Metaphysis	5.0	12	Bioglass	-	500	76.0	Micro-CT	80.0
Su et al. 20	Rabbit	Metaphysis	5.0	12	Bioglass	-	500	76.0	Micro-CT	48.0
Zhao et al. 21	Rat	Calvaria	5.0	8	HA	BMP-2	450	75.0	Micro-CT	43.0
Zhao et al. 21	Rat	Calvaria	5.0	8	HA	BMP-2	450	75.0	Micro-CT	27.0
Zhao et al. 21	Rat	Calvaria	5.0	8	HA	-	450	75.0	Micro-CT	10.0
Klijn et al. 22	Rat	Calvaria	6.0	12	CP	-	500	53.2	HMM	10.8
Klijn et al. 22	Rat	Calvaria	6.0	12	CP	-	500	44.5	HMM	7.0
Klijn et al. 22	Rat	Calvaria	6.0	12	CP	-	500	42.0	HMM	6.8
Liu et al. 23	Rat	Calvaria	4.6	24	Bioglass	-	150	50.0	HMM	24.0
Liu et al. 23	Rat	Calvaria	4.6	24	Bioglass	-	500	80.0	HMM	36.0
Tayton et al. 24	Goat	Metaphysis	8.0	13	HA	BMSCs	192	63.4	Micro-CT	38.1
Tayton et al. 24	Goat	Metaphysis	8.0	13	HA	-	192	63.4	Micro-CT	24.8

NBF, new bone formation; HA, hydroxyapatite; CP, calcium phosphate; CS, calcium silicate; FGF, fibroblast growth factor; BMP-2, bone morphogenetic protein-2; BMSC, bone marrow mesenchymal stem cells; HMM, histomorphometry.

**Table 2:** Median, minimum and maximum values for NBF rates and the experimental group counts according to the experimental model and biomaterial characteristics.

	HMM			Micro-CT		
	NBF	Count	p-value	NBF	Count	*p*-value
**Animal**						
Rat	16.92 (6.80-36.00)	5	0.377	26.27 (10.00-43.00)	6	0.340
Goat	20.67 (0.00-37.00)	6		31.45 (24.80-38.10)	2	
Rabbit	28.04 (17.00-60.00)	9		46.65 (15.60-80.00)	4	
**Region**						
Calvaria	18.53 (6.80-36.00)	9	0.102	26.67 (10.00-43.00)	3	0.459
Metaphisis	26.75 (0.00-60.00)	11		36.34 (15.60-80.00)	9	
**Basic material**						
CP	16.87 (0.00-37.00)	12	NT	15.60 (15.60-15.60)	1	NT
HA	27.55 (23.00-32.20)	4		29.28 (10.00-43.00)	9	
CS	28.36 (28.36-28.36)	1		-	0	
Glass	30.00 (24.00-36.00)	2		80.00 (48.00-80.00)	2	
HA+CP	60.00 (60.00-60.00)	1	-	-	0	
**Osteoinductor**						
None	23.05 (0.00-60.00)	20	NT	34.54 (10.00-80.00)	7	0.626
FGF	-	0		28.60 (28.50-28.70)	2	
BMP-2	-	0		35.00 (27.00-43.00)	2	
BMSCs	-	0		38.10 (38.10-38.10)	1	
**Maximum pore size**						
< 300 μm	20.13 (0.00-37.00)	8	0.588	31.45 (24.80-38.10)	2	0.829
300 - 500 μm	25.50 (6.80-60.00)	10		34.42 (10.00-80.00)	10	
> 500 μm	22.50 (20.00-25.00)	2		-	-	
**Porosity**						
< 5%	11.50 (0.00-32.20)	4	0.035	-	-	
5% - 75%	21.50 (10.80-28.36)	10		28.10 (20.40-38.10)	5	0.016
> 75%	33.33 (20.00-60.00)	6		38.09 (10.00-80.00)	7	
**Total**	**23.05 (0.00-60.00)**	**20**		**33.93 (10.00-80.00)**	**12**	

NBF, new bone formation; HA, hydroxyapatite; CP, calcium phosphate; CS, calcium silicate; FGF, fibroblast growth factor; BMP-2, bone morphogenetic protein-2; BMSC, bone marrow mesenchymal stem cells; HMM, histomorphometry; NT, not tested. P-values from Kruskal –Wallis test.
